# Short-term indoor fine particulate matter exposure impedes cognitive performance and autonomic nervous system in adults

**DOI:** 10.1016/j.isci.2026.117904

**Published:** 2026-10-15

**Authors:** Jiaxu Zhou, Gesche Huebner, Kathy Y. Liu, Marcella Ucci

**Affiliations:** 1 UCL Institute for Environmental Design and Engineering (IEDE), The Bartlett, University College London (UCL), Central House, 14 Upper Woburn Place, London WC1H 0NN, UK; 2 School of Architecture, Inner Mongolia University of Technology (IMUT), Hohhot, Inner Mongolia 010051, China; 3 European Centre for Environment & Human Health (ECEHH), University of Exeter, Peter Lanyon Building 12, Penryn, Cornwall TR10 8RD, UK; 4 Division of Psychiatry, UCL Faculty of Brain Sciences, University College London (UCL), 149 Tottenham Court Road, London W1T 7NF, UK

**Keywords:** indoor air quality, fine particulate matter, cognition, autonomic nervous system, healthy and sustainable environment

## Abstract

Evidence suggests short-term exposure to fine particulate matter (PM2.5) air pollution can impact health, yet its wider cognitive effects and underlying mechanisms are unknown. In this randomized single-blind crossover lab-based study, 90 working-age adults were exposed to two stable indoor PM2.5 conditions generated by candle-burning: low concentration (1.70 μg/m^3^, representing clean air) achieved via an air purifier, and high concentration (31.22 μg/m^3^, representing global average exposure) using a sham filter in the purifier. Participants’ cognitive performance across various domains was assessed, alongside heart rate variability (HRV) and electrodermal activity (EDA) to appraise potential mediating mechanisms of autonomic nervous activity on cognition. Higher PM2.5 exposure resulted in lower HRV and poorer cognitive performance in attention and memory, but not in coordination or reasoning. Decreased HRV partly mediated declines in some cognitive functions. The findings could have broader implications for workforce productivity beyond direct effects on ill health.

## Highlights

• This is a randomized, single-blind crossover lab-based study
• Short-term indoor PM2.5 exposure impedes cognitive and autonomic function in adults
• PM2.5 effects on executive skills are partly mediated through cardiovagal responses
• Hidden cognitive effects of PM2.5 may affect workforce productivity beyond ill health

## Graphical abstract

**Figure:**
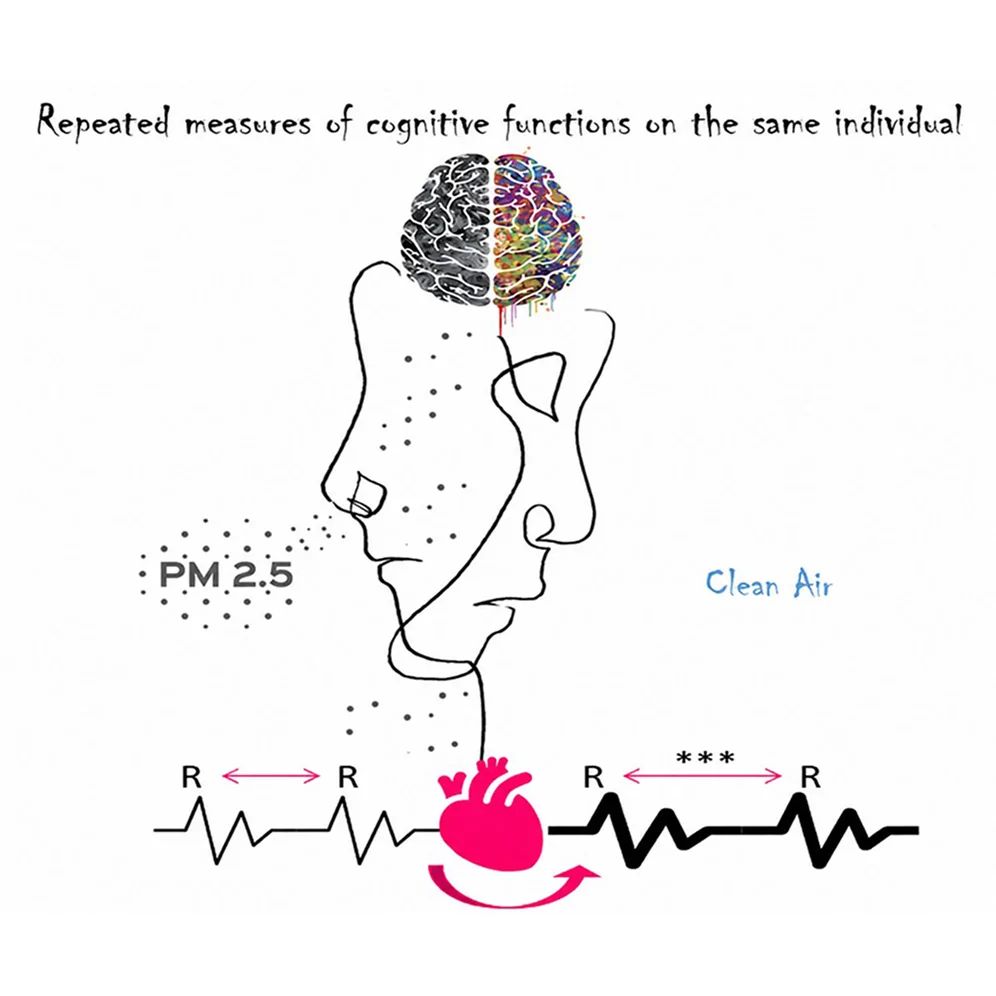

## Teaser

Cognitive neuroscience; Pollution

## Introduction

In global urbanized and industrialized modern societies, people spend more than 90% of their time indoors and hence indoor air can impact human health.^1^ Recent decades have seen tremendous growth of studies on the long-term health effects of particulate matter (PM) air pollution, including emerging evidence on risks of developing cognitive problems and mental health issues.^2^^,^^3^^,^^4^ Moreover, 99% of the world’s population live in places with unhealthy air,^5^ further emphasizing the need for addressing air quality issues both indoors and outdoors and their pervasive impact on health.

Levels of annual average fine PM, i.e., PM2.5, which has a diameter less than or equal to 2.5 μm, have dramatically reduced over the past few decades through large-scale emission control policies, as reported in the Chief Medical Officer’s Annual Report 2022: Air Pollution.^6^ However, even in some areas where air pollution levels are within the WHO-recommended annual level of PM2.5 of 5 μg/m^3^, short-term (hours to days) peaks in PM2.5 air pollution are frequently reported and are linked to human mortality and morbidity.^7^^,^^8^^,^^9^ While policy initiatives from government and non-governmental organizations—such as net zero targets, emission control regulations, and Clean Air Acts—have been implemented to reduce air pollution, specific recommendations for indoor PM2.5 limits related to human cognition and mental health risks remain absent, due to limited evidence. Cost-effective approaches and interventions against the potential detrimental impacts of indoor PM2.5 are critical and hold high public health value.^10^ Understanding the impacts on cognition of short-term fine PM exposure in indoor environments is quintessentially important for working-age adults who comprise more than 60% of the global population.^11^

Emerging evidence suggests that exposure to PM2.5 air pollution may worsen cognitive decline and impairment.^10^^,^^12^^,^^13^^,^^14^^,^^15^ However most studies focus on older adults (over 50 years) and are based on ambient exposures over medium-long term timescales (i.e., one month to years). Overall, however, the evidence is heterogeneous and not fully conclusive.^16^ Research into the short-term cognitive effects of PM2.5 is limited, with most studies focusing on only one or two cognitive tasks,^17^^,^^18^^,^^19^ limiting their generalizability, particularly for exposures occurring indoors. The mixed findings to date may be due to differences in the outcomes evaluated (i.e., specific cognitive skills or domains) and to the predominance of observational study designs, which are unlikely to sufficiently control air quality exposure alongside other environmental factors. To date, there is underlying epidemiological evidence and confidence in the estimate that air pollution is likely associated with cognitive decline,^20^ but there is limited comprehensive analysis or evidence on which cognitive domains are more or less impacted by PM2.5 exposure and associated pathways in working-age adults. Recent experimental evidence^21^ suggests that PM exposure disrupts attention and emotional recognition through lung-brain mechanisms, which are hypothesized to involve either direct or indirect pathways. Furthermore, a comprehensive report from the committee on the medical effects of air pollutants,^20^ reviewing broader epidemiological evidence, indicates that PM exposure is causally associated with cardiovascular conditions, including cerebrovascular disease. The associations between PM exposure and the onset and progression of cognitive decline are likely to be, at least partially, secondary to these cardiovascular effects (ibid). However, to date the potential indirect pathways through which air pollutants such as PM2.5 may elicit cognitive decline remain poorly understood. Nevertheless, our previous real-world study represents one of the few attempts to comprehensively address a broad range of cognitive domains^22^ and physiological markers for indirect pathways via cardiac autonomic nervous system.^23^ Although providing valuable insights under realistic conditions, these studies had limited control over potentially confounding environmental parameters.

To help address these knowledge gaps, a lab-based study was conducted, aiming to investigate the impact of indoor PM2.5 exposure on cognition across a wide range of cognitive domains in working-age adults. We hypothesized that elevated short-term indoor PM2.5 exposure negatively affects cognitive performance. Given the direct effects of PM2.5 on cardiorespiratory function,^24^^,^^25^ and established connection between physiological signals and autonomic system activity on cognition,^26^^,^^27^ we also hypothesized that autonomic nervous system changes may mediate any impact of short-term PM2.5 exposure on cognition. We measured participants’ physiological responses, heart rate variability (HRV) and electrodermal activity (EDA). EDA, a measure of sweat-induced changes in skin conductance, is proposed to index sympathetic control.^28^ HRV, the beat-to-beat variation in heart rate, is proposed to index cardiac parasympathetic (vagal) control.^29^^,^^30^ Generally, higher HRV indicates better cardiovagal control and self-regulatory and adaptive capacity, and is associated with better mental and physical health.^31^^,^^32^^,^^33^

The study used a randomized single-blind crossover design with repeated measures, conducted in an environmentally controlled climate chamber in London, UK. Indoor PM2.5 was generated by combustion of non-scented candles in both conditions. Candles are used in many situations on up to a daily basis, for example, for lighting, religious or spiritual purposes and relaxation. They are a well-known source of PM2.5 and have been used in a previous relevant study.^17^ An identical candle-burning setup was used across both conditions. A high exposure condition was achieved in the environmentally controlled climate chamber, with a stable PM2.5 concentration of 31.2 μg/m^3^, which is similar to the global average PM2.5 exposure of 31.3 μg/m^3^ reported in the *State of Global Air 2024*.^5^ Air filtration was used to achieve a low exposure condition, with a reduced PM2.5 level of 1.70 μg/m^3^, which is well below the yearly and 24 h recommendations (respectively 5 μg/m^3^ and 15 μg/m^3^) from the *WHO Global Air Quality Guidelines*.^34^ Specifically, a portable air purifier was present and turned on in both conditions; however, in the high condition a sham filter was used to ensure participants would be blind to the experimental condition, while in the low condition a high efficiency particulate air (HEPA) filter filtered out PM. Key environmental parameters, including ventilation rate, relative humidity, and air temperature, were precisely controlled and kept consistent across conditions by the climate chamber system.

The study included 90 working-age adults, a sample size based on statistical power calculation (*See* STAR Methods *section*), who were randomized to a sequence of two exposure conditions, either high-low or low-high. During each exposure condition which lasted approximately 1.5 h, participants’ cognitive performance was evaluated using the general cognitive assessment (with a parallel version for each condition), a validated computer-based neurological test battery (CogniFit Inc., San Francisco, USA). Autonomic nervous system responses including EDA and HRV were collected before and during cognitive testing. In addition to reporting the effects of PM2.5 on cognitive performance, we performed a mediation analysis to understand if the effects of PM2.5 on cognition are transmitted via changes in cardiovagal control as indexed by HRV.

## Results

The average PM2.5 exposure in the high and low conditions were 31.22 μg/m^3^ (SD = 7.30) and 1.70 μg/m^3^ (SD = 1.30), respectively. Other indoor environment parameters (relative humidity, TVOCS, CO2, ventilation rate, and temperature) remained stable across the two conditions (Table 1). In addition, as the mass concentrations of PM1, PM2.5, and PM10 were almost identical, this suggests that the PM in the chamber was predominantly fine PM, most of which is smaller than 2.5 μm. While a statistically significant difference in CO_2_ levels was found between the high and low PM2.5 exposure conditions, a difference of 63 ppm could be considered of limited practical impact in the context of the indoor environment from human health, comfort, and cognition perspectives.^35^^,^^36^ There was no significant difference in sleep hours, coffee and meal consumption status across the two conditions. 64.4% of the 90 participants were female. All subjects were young adults aged 18–40 years with a master’s degree or above, with the majority of participants (88.9%) within the 18–30 age band (see Table 2).

**Table 1:** Environment and participant-level information

	Condition		
	High exposure to fine PM	Low exposure to fine PM	
Indoor environment level information^a^	Mean (SD)	Mean (SD)	Paired *t* test
Fine particulate matter (PM)			
PM1 (μg/m^3^)	30.7 (6.9)	1.69 (1.2)	*p* < 0.001
PM2.5 (μg/m^3^)	31.2 (7.3)	1.70 (1.3)	*p* < 0.001
Environmental parameters			
Relative humidity (%)	51.1 (2.9)	51.75 (0.3)	*p* = 0.052
Air temperature (°C)	23.3 (0.3)	23.34 (0.3)	*p* > 0.1
CO_2_ (ppm)	873.2 (54.1)	809.81 (63.0)	*p* < 0.001
TVOCS (μg/m^3^)	11.44 (0.9)	11.16 (1.7)	*p* > 0.1

Participant level information	Answers	N (%)	N (%)	Chi-square test
Sleep hours	<7 h	31 (34.4%)	28 (31.1%)	*p* > 0.1
	7–9 h	57 (63.3%)	58 (64.4%)	
	>9 h	2 (2.2%)	4 (4.4%)	
Coffee status	yes	21 (23.3%)	16 (17.8%)	*p* > 0.1
	no	69 (76.7%)	74 (82.2%)	
Meal status	yes	75 (83.3%)	74 (82.2%)	*p* > 0.1
	no	15 (16.7%)	16 (17.8%)	

PM1, particulate matter with an aerodynamic diameter of ≤1 μm; PM2.5, particulate matter with an aerodynamic diameter of ≤2.5 μm.

a Indoor environmental parameters were collected in a 60 m^3^ climate Chamber with 108 m^3^/h ventilation rate.

**Table 2:** Demographics

	Answers	N (%)
Total participants		90
Sex	male	32 (35.6)
	female	58 (64.4)
Age band	18–30	80 (88.9)
	31–40	10 (11.1)
Education level	bachelor	1 (1.1)
	master and above	89 (98.9)

### Effects of indoor fine particulate matter on cognition

We first determined the impacts of indoor PM2.5 levels on cognitive performance by treating the experimental PM2.5 condition as a categorical predictor variable (i.e., high or low) (*n* = 90) in mixed effects linear regression model adjusted for sex. The average values of each task and metric are presented in Figures 1 and 2, and findings pertaining to all statistical outcomes are presented in Table 3. The findings revealed that, compared with the high indoor PM2.5 condition, participants who were in a low PM2.5 environment exhibited significantly better cognitive performance in a number of tasks as measured by higher accuracy and/or faster reaction times for correct responses.

**Figure 1:**
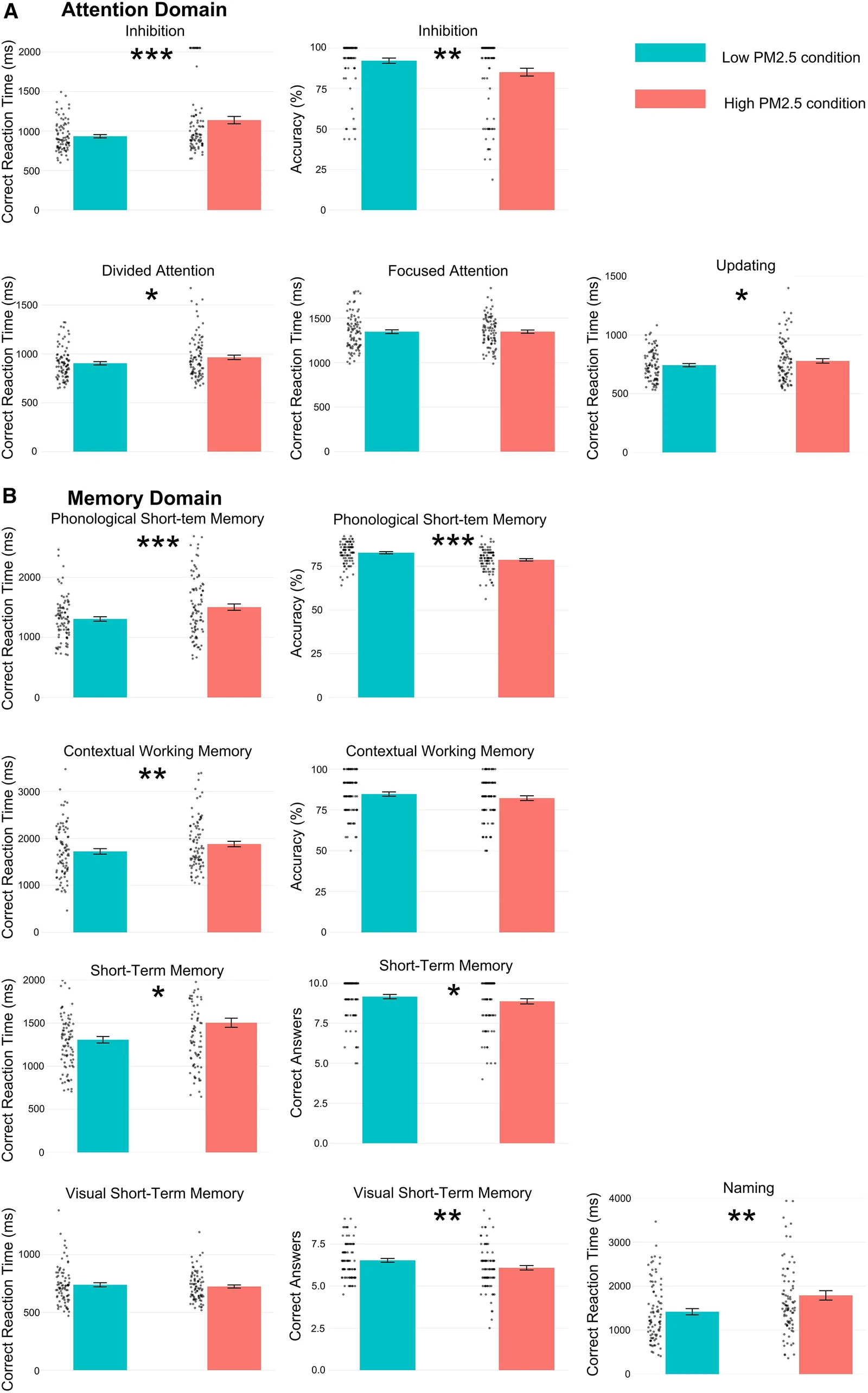
Cognitive performance across attention (A) and memory (B) under high and low PM2.5 exposure conditions (*n* = 90). Error bars represent standard error (±SE). Statistical significance was assessed using a generalized linear mixed-effects regression model adjusted for sex. Asterisks indicate *p* values: ∗*p* < 0.05, ∗∗*p* < 0.01, and ∗∗∗*p* < 0.001.

**Figure 2:**
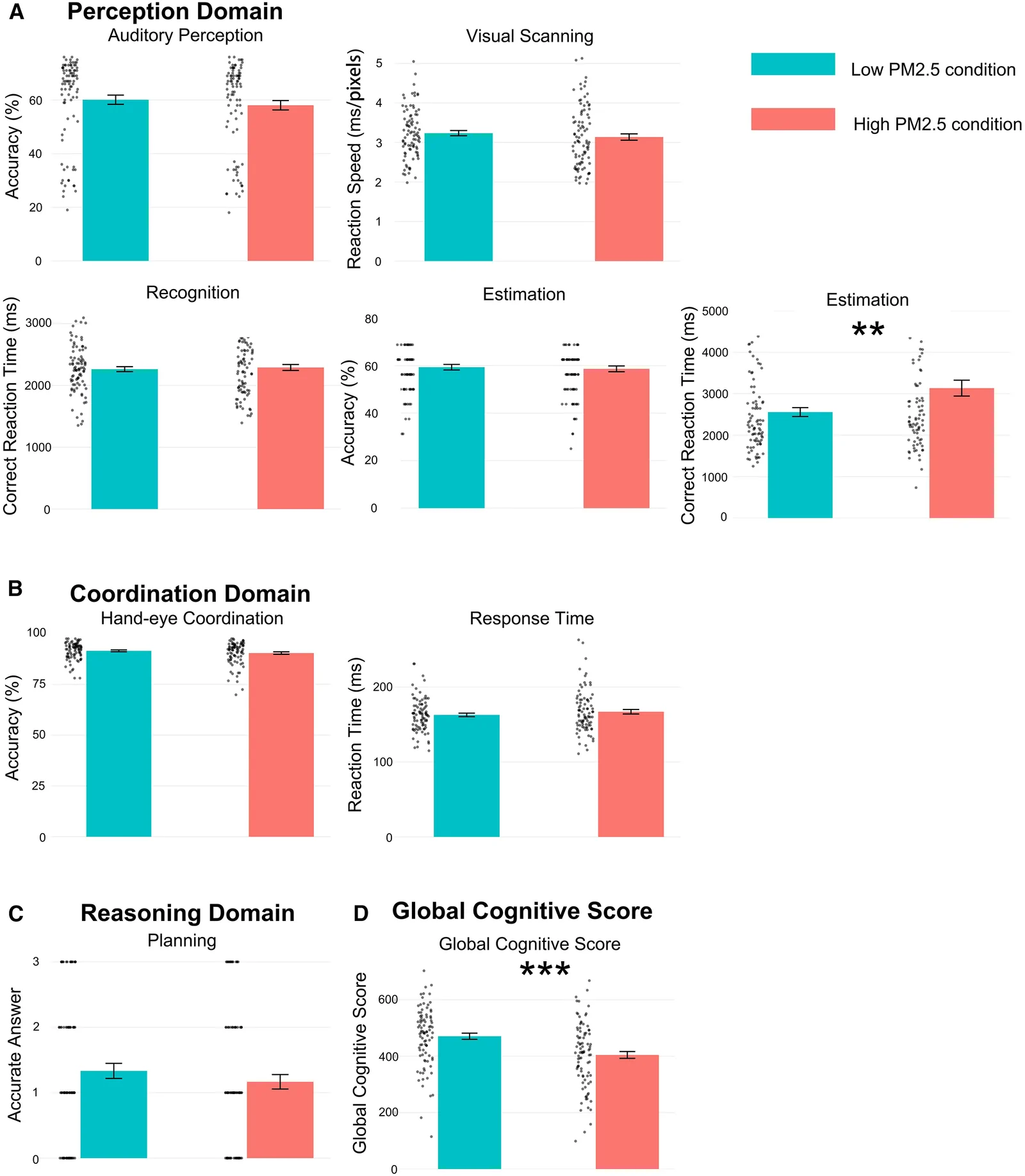
Cognitive performance across perception (A), coordination (B), reasoning (C), and global cognitive score (D) under high and low PM2.5 exposure conditions (*n* = 90). Error bars represent standard error (±SE). Statistical significance was assessed using a generalized linear mixed-effects regression model adjusted for sex. Asterisks indicate *p* values: ∗*p* < 0.05, ∗∗*p* < 0.01, and ∗∗∗*p* < 0.001.

**Table 3:** Statistical analysis of the cognitive performance in the experiment (*n* = 90), with low PM2.5 condition as the reference category, and the β coefficients were adjusted for sex

Domain	Skill	Metric	β (95% CI)	*p* value	Cohen’s d
Memory	phonological short-term memory	correct reaction time (Milliseconds)	199.95 (105.80, 293.81)	*p* < 0.001	0.455
		accuracy (%)	−4.01 (5.50–2.53)	*p* < 0.001	0.650
	contextual working memory	correct reaction time (milliseconds)	154.83 (46.34, 263.48)	*p* = 0.006	0.287
		accuracy (%)	−2.50 (−5.92,0.91)	*p* = 0.152	0.192
	short-term memory	correct reaction time (illiseconds)	43.60 (10.09,76.52)	*p* = 0.011	0.128
		correct answers (10 answers in total)	−0.30 (−0.54, −0.05)	*p* = 0.019	0.211
	visual short-term memory	correct reaction time (milliseconds)	−13.29 (−42.63 15.91)	*p* = 0.374	0.101
		correct answers (10 answers in total)	−0.44 (−0.69, −0.20)	*p* = 0.001	0.379
	naming	correct reaction time (milliseconds)	368.94 (152.60, 586.31)	*p* = 0.001	0.436
Attention	divided attention	correct reaction time (milliseconds)	61.16 (14.39,107.93)	*p* = 0.012	0.324
	inhibition	correct reaction time (milliseconds)	203.03 (115.28, 291.40)	*p* < 0.001	0.597
		accuracy (%)	−7.01 (−12.18, −1.86)	*p* = 0.009	0.364
	focused attention	correct reaction time (milliseconds)	0.767 (−47.65, 49.18)	*p* = 0.975	0.004
	updating	correct reaction time (milliseconds)	37.03 (7.03, 66.98)	*p* = 0.017	0.233
Perception	auditory perception	accuracy (%)	−2.25 (−5.53,1.04)	*p* = 0.180	0.126
	estimation	correct reaction time (milliseconds)	581.18 (162.05, 997.59)	*p* = 0.007	0.400
		accuracy (%)	−0.69 (−3.96, 2.58)	*p* = 0.677	0.061
	recognition	correct reaction time (milliseconds)	27.97 (−51.20, 107.10)	*p* = 0.488	0.065
	visual scanning	reaction speed (milliseconds/pixels)	−0.10 (−0.29,0.10)	*p* = 0.320	0.142
Coordination	hand-eye coordination	accuracy (%)	−1.09 (−2.27, 0.08)	*p* = 0.071	0.221
	response time	reaction time (milliseconds)	4.36 (−0.64, 9.35)	*p* = 0.089	0.166
Reasoning	planning	correct answers (3 answers in total)	−0.17 (−0.42, 0.08)	*p* = 0.195	0.156

In the memory domain, a consistent effect was observed across five skills (phonological short-term memory, contextual working memory, naming, visual short term memory, and short term memory), where high levels of indoor PM2.5 exposure led to slower reaction times and/or decreased accuracy, with effect size Cohen’s d ranging from 0.128 to 0.650. In the attention domain, high levels of indoor PM2.5 exposure led to worse performance, particularly in tasks on divided attention, inhibition, and updating (but not focused attention), with effect size Cohen’s d ranging from 0.233 to 0.597 in the tasks with significant effects. In the perception domain, only the estimation task showed slower correct reaction time under the high PM2.5 condition, whereas the other three tasks (auditory perception, visual scanning, recognition, and estimation) showed non-significant differences across the two conditions. Coordination and reasoning domains showed no significant differences between conditions. See Table 3 for all statistical details. The *p* values on these variables were robust to multiple hypothesis testing after false discovery rate correction (FDR *p* < 0.05).^37^ See Table S5.

We additionally determined the impacts of indoor PM2.5 levels on global cognitive scores (GCS). The score of GCS is calculated by the CogniFit program^38^^,^^39^ and represents an aggregation of performance on the different tasks, ranging from 0 to 800 with higher values indicating better performance. The results indicated that compared to the high PM2.5 condition (mean = 404.51, SD = 114.09), individuals performed better in the low PM2.5 condition (mean = 470.84, SD = 103.09) (β = 66.33, *p* < 0.001, 95% CI [44.539, 88.128]), with effect size Cohen’s d = 0.60, indicating a moderate effect. Furthermore, no evidence of carry-over effect was observed in this study. The mean summed GCS across two conditions were 871.36 and 879.36 in the two exposure-sequence groups, respectively, with no statistically significant difference (mean difference = −8.00, 95% CI [−88.93, 72.93], *p* > 0.1).

The analysis revealed significant sex difference effects for only four tasks (*p* < 0.05). Specifically, for visual short-term memory, females exhibited slower correct reaction times than males (β = 61.77, 95% CI [6.38, 117.15], *p* = 0.0317) and lower accurate answers (β = −0.5449, 95% CI [−0.9724, −0.1173], *p* = 0.0144). For hand-eye coordination, females showed lower accuracy (β = −3.0495, 95% CI [−4.7498, −1.3456], *p* = 0.0007). For response time, females had longer reaction times than males (β = 11.784, 95% CI [2.314, 21.257], *p* = 0.0168).

### Effects of indoor fine particulate matter on autonomic nervous system indices

The effects of indoor fine PM air pollution on HRV indices are shown in Table 4 across the high and low PM2.5 conditions (*n* = 79). The indices, including standard deviation of the normal-to-normal intervals (SDNN), percentage of consecutive RR intervals that differ by more than 50 ms (pNN50), root-mean-square of successive differences (RMSSD), and high-frequency power (HF), are presented. The findings revealed that high levels of PM2.5 significantly decreased the fixed effect of PM2.5 on all HRV indices during, but not before, the cognitive testing period (i.e., when there was a cognitive demand). The changes in HRV indices were moderate in magnitude with Cohen’s d varying from 0.40 to 0.55.

**Table 4:** Statistical analysis of HRV parameters in the experiment (*n* = 79), with the low PM2.5 exposure condition as the reference category. HRV parameters were log10-transformed for the analyses, and the β coefficients were adjusted for sex

Period	Condition	SDNN	RMSSD	pNN50	HF
		β (95% CI), *p* value	β (95% CI), *p* value	β (95% CI), *p* value	β (95% CI), *p* value
Pre-testing period	low PM2.5 condition	Ref.	Ref.	Ref.	Ref.
	high PM2.5 condition	−0.018 (−0.081, 0.045), *p* = 0.569	−0.021 (−0.099, 0.056), *p* = 0.589	−0.073 (−0.212, 0.066), *p* = 0.302	−0.032 (−0.092, 0.028), *p* = 0.294
	low PM2.5 condition mean (SD)	81.79 (60.83)	78.95 (78.00)	21.50 (17.65)	0.40 (0.14)
	high PM2.5 condition mean (SD)	79.45 (63.38)	73.76 (65.79)	20.94 (16.56)	0.39 (0.17)
Cognitive testing period	low PM2.5 condition	Ref.	Ref.	Ref.	Ref.
	high PM2.5 condition	−0.114 (−0.154, −0.074), *p* < 0.001	−0.141 (−0.194, −0.088), *p* < 0.001	−0.129 (−0.198, −0.060), *p* < 0.001	−0.088 (−0.123, −0.053), *p* < 0.001
	low PM2.5 condition mean (SD)	118.19 (68.32)	117.95 (86.39)	29.65 (18.28)	0.36 (0.11)
	high PM2.5 condition mean (SD)	87.14 (45.40)	78.94 (50.64)	23.03 (14.75)	0.31 (0.12)
	Cohen’s d	0.53	0.55	0.40	0.52

SDNN: standard deviation of normal-to-normal intervals in milliseconds (ms); RMSSD: root-mean-square of the successive differences in milliseconds (ms); pNN50: Percentage of successive R-R intervals that differ by more than 50 ms in percentage (%); HF: normalized high-frequency spectral power in normalized units (nu).

In secondary sensitivity analyses, the models were additionally adjusted for pre-cognitive testing HRV scores (i.e., the 5 min prior to the cognitive testing), and the results remained significantly higher in the low PM2.5 exposure condition compared to the high exposure condition for SDNN (β = 0.106, 95% CI [0.060,0.151], *p* < 0.001), RMSSD (β = 0.128 [95% CI: 0.068, 0.189], *p* < 0.001), pNN50 (β = 0.070 [95% CI: 0.015, 0.125], *p* = 0.013), and HF (β = 0.082 [95% CI: 0.045, 0.118], *p* < 0.001).

The sex difference effects on the HRV outcomes were not significant in any model at the period before the cognitive testing (*p* > 0.1). During cognitive testing, males exhibited significantly lower pNN50 compared to females (β = −0.240, 95% CI [−0.408, −0.072], *p* = 0.005). Other HRV metrics did not show a significant sex difference effect (*p* > 0.1).

For EDA (*n* = 79), we found no significant differences between the two exposure conditions in terms of skin conductance level (SCL) and skin conductance response (SCR). Specifically, during the resting period before cognitive testing, the results for SCL (β = −0.018, 95% CI [−0.094, 0.058], *p* = 0.635) and SCR (β = −0.108, 95% CI [−0.247, 0.031], *p* = 0.127) indicated no significant effects. Similarly, during cognitive testing, both SCL (β = −0.016, 95% CI [−0.101, 0.069], *p* = 0.709) and SCR (β = −0.074, 95% CI [−0.221, 0.072], *p* = 0.318) showed no significant differences.

Regarding sex differences in EDA measures before cognitive testing, males exhibited significantly higher SCL (β = 0.216, 95% CI [0.056, 0.377], *p* = 0.009) and SCR (β = 0.303, 95% CI [0.013, 0.593], *p* = 0.040) compared to females. During cognitive testing, males continued to show higher SCL (β = 0.194, 95% CI [0.043, 0.344], *p* = 0.012), but no significant difference in SCR (β = 0.160, 95% CI [−0.064, 0.384], *p* = 0.161). After adjusting for pre-cognitive testing EDA, the effect of sex differences on SCL (β = 0.039, 95% CI [−0.065, 0.143], *p* = 0.462) and SCR (β = −0.014, 95% CI [−0.172, 0.144], *p* = 0.858) were not significant.

### Mediation effect of autonomic nervous system

Mediation analysis, adjusted for sex, was conducted to investigate whether autonomic nervous system responses (measured by HRV) mediated the observed associations between indoor PM2.5 exposure and cognitive performance. RMSSD and HF are highly correlated and are proposed to reflect parasympathetic influence, with RMSSD being less sensitive to respiratory effects,^40^ whereas SDNN represents both sympathetic and parasympathetic influences on the heart.^30^Therefore, we selected two time-domain HRV measures, SDNN and RMSSD, to examine the influence of both aspects of the autonomic nervous system.^32^^,^^41^^,^^42^

The mediation analysis results suggested that HRV had varying mediation effects on the impact of high indoor PM2.5 exposure on poorer cognitive task performance. Notably, significant mediation effects were observed in the inhibition and phonological short memory task performance. Specifically, high PM2.5 levels led to slower inhibition task correct reaction times and lower phonological short-term memory accuracy through lower SDNN. A bootstrap CI for the specific average causal mediation effect (ACME) did not include zero, providing evidence for statistically significant mediation, for inhibition task correct reaction time (ACME = 33.26, 95% Boot CI [7.04, 72.41]) and for phonological short-term memory task accuracy (ACME = −0.59, 95% Boot CI [−1.37,−0.04]. Similarly, the RMSSD mediation showed similar results for inhibition task correct reaction time (ACME = 24.04, 95% Boot CI [2.12, 56.29]). Details for all mediation models are included in Table 5.

**Table 5:** Mediation effect of variables on the relationship between PM2.5 exposure, HRV, and task performance (*n* = 79)

Cognitive function			Mediator: SDNN (*n* = 79)			Mediator: RMSSD (*n* = 79)		
Domain	Skill	Metric	Total effect (95% bootstrap CI)	Average direct effect (95% bootstrap CI)	Average causal mediation effect (95% bootstrap CI)	Total effect (95% bootstrap CI)	Average direct effect (95% bootstrap CI)	Average causal mediation effect (95% bootstrap CI)
Attention	divided attention	correct reaction time (milliseconds)	61.15 (7.03, 127.28)	52.49 (−3.36, 119.34)	8.66 (−5.33, 27.31)	61.15 (2.86, 120.92)	54.87 (−4.58, 114.25)	6.28 (−9.64, 24.38)
	inhibition	correct reaction time (milliseconds)	177.99 (80.59, 273.25)	144.73 (47.93, 237.08)	**33.26**^a^**(7.04, 72.41)**	177.99 (79.71, 289.79)	153.95 (62.28, 260.99)	**24.04**^a^**(2.12, 56.29)**
		accuracy (%)	−6.09 (−12.32, −0.74)	−5.26 (−11.08, −0.11)	−0.831 (−2.71, 0.84)	−6.09 (−12.02, −0.48)	−5.67 (−11.34, −0.38)	−0.42 (−2.01, 1.13)
	sustained attention	correct reaction time (milliseconds)	6.04 (−53.32, 65.40)	11.95 (−49.55, 73.44)	−5.91 (−21.57, 11.95)	6.04 (−53.32, 65.40)	13.29 (−47.86, 74.44)	−7.25 (−22.43, 8.77)
	updating	correct reaction time (milliseconds)	32.23 (−11.82, 78.63)	31.17 (−14.09, 78.02)	1.06 (−10.69, 13.14)	32.23 (−14.02, 78.75)	29.97 (−16.38, 74.68)	2.27 (−7.62, 13.17)
Memory	visual short-term memory	correct reaction time (milliseconds)	−20.66 (−69.06, 23.71)	−15.78 (−63.69, 28.84)	−4.88 (−18.24, 7.10)	−20.66 (−69.66, 23.90)	−16.21 (−65.79, 26.43)	−4.45 (−17.26, 6.38)
		correct answers (10 answers in total)	−0.40 (−0.77, −0.05)	−0.48 (−0.84, −0.11)	0.072 (−0.01, 0.19)	−0.40 (−0.76, −0.08)	−0.47 (−0.83, −0.14)	0.07 (−0.01, 0.18)
	phonological short-term memory	correct reaction time (milliseconds)	183.92 (49.57, 313.23)	158.00 (23.81, 287.95)	25.92 (−3.97, 76.17)	183.92 (61.09, 319.19)	156.1681 (22.75, 287.79)	27.75 (−3.31, 71.54)
		accuracy (%)	−3.83 (−5.70, −1.96)	−3.24 (−5.01, −1.31)	**−0.59**^a^**(−1.37, −0.04)**	−3.83 (−5.95, −1.97)	−3.33 (−5.46, −1.37)	−0.50 (−1.15, 0.00)
	contextual working memory	correct reaction time (milliseconds)	131.78 (−40.48, 301.64)	118.54 (−60.68, 297.18)	13.24 (−26.11, 58.07)	131.78 (−28.06, 297.75)	122.60 (−44.97, 299.54)	9.18 (−29.09, 54.07)
		accuracy (%)	−2.53 (−6.65, 1.42)	−1.54 (−5.87, 2.63)	−0.99 (−2.75, 0.04)	−2.53 (−6.48, 1.49)	−1.60 (−5.84, 2.58)	−0.93 (−2.54, 0.10)
	short-term memory	correct reaction time (milliseconds)	16.12 (−62.90, 79.96)	19.76 (−46.32, 77.32)	−3.64 (−25.77, 14.18)	16.12 (−57.40, 84.25)	21.10 (−39.69, 84.29)	−4.98 (−26.00, 9.01)
		correct answers (10 answers in total)	−0.25 (−0.68, 0.18)	−0.34 (−0.78, 0.12)	0.09 (−0.01, 0.23)	−0.25 (−0.71, 0.18)	−0.34 (−0.82, 0.11)	0.10 (0.00, 0.24)
	naming	correct reaction time (milliseconds)	303.89 (53.81, 576.85)	290.85 (32.43, 572.97)	13.05 (−49.70, 81.59)	303.89 (57.30, 549.88)	300.83 (35.64, 559.35)	3.06 (−52.49, 61.26)
Perception	estimation	correct reaction time (milliseconds)	399.32 (−51.94, 860.65)	378.71 (−83.55, 842.60)	20.61 (−88.48, 147.87)	399.32 (−46.17, 820.48)	378.72 (−61.85, 800.10)	20.61 (−97.36, 141.61)
		accuracy (%)	0.24 (−3.15, 3.88)	0.81 (−2.58, 4.71)	−0.57 (−1.72, 0.32)	0.24 (−3.22, 3.50)	0.78 (−2.96, 4.18)	−0.54 (−1.69, 0.27)
	auditory perception	accuracy (%)	−1.18 (−6.62, 3.98)	−1.07 (−6.62, 4.05)	−0.11 (−1.63, 1.22)	−1.18 (−6.41, 3.75)	−1.49 (−6.77, 3.73)	0.32 (−0.94, 1.78)
	recognition	correct reaction time (milliseconds)	18.52 (−116.47, 150.21)	22.12 (−116.33,155.59)	−3.60 (−44.02, 30.36)	18.52 (−109.51, 151.88)	26.01 (−105.70, 155.47)	−7.49 (−43.35, 25.74)
	visual scanning	reaction speed (milliseconds/pixels)	−0.09 (−0.31, 0.10)	−0.03 (−0.26, 0.16)	−0.059 (−0.14, 0.00)	−0.09 (−0.31, 0.10)	−0.04 (−0.25, 0.16)	−0.05 (−0.14, 0.00)
Coordination	hand-eye coordination	accuracy (%)	−1.28 (−2.96, 0.13)	−1.11 (−2.90, 0.54)	−0.17 (−0.66, 0.27)	−1.28 (−2.73, 0.15)	−1.16 (−2.75, 0.38)	−0.13 (−0.55, 0.32)
	response time	reaction time (milliseconds)	5.14 (−2.85, 13.01)	5.68 (−3.72, 14.51)	−0.54 (−3.02, 2.13)	5.14 (−2.53, 13.40)	4.89 (−3.48, 13.70)	0.25 (−1.89, 2.80)
Reasoning	planning	correct answers (3 answers in total)	−0.17 (−0.51, 0.15)	−0.16 (−0.50, 0.15)	−0.001 (−0.09, 0.08)	−0.17 (−0.49, 0.16)	−0.17 (−0.50, 0.17)	0.008 (−0.08, 0.10)

a Mediation effect significance at 0.05 level. A bootstrap CI for the specific average causal mediation effect (indirect effect), did not include zero providing evidence for statistically significant mediation.

## Discussion

To our knowledge, this is the first lab-based study conducted in an environmentally controlled climate chamber to examine the impact of indoor short-term PM2.5 exposure at relatively stable levels on a wide range of cognitive domains in adults, while precisely controlling for variations in other environmental factors. Our experiment demonstrates that indoor short-term exposure to high levels (31.22 μg/m^3^) of PM2.5 impedes performance across multiple cognitive domains in working-age participants. We also demonstrate that exposure to these high levels was associated with lower HRV during cognitive testing, which mediated the effect of PM2.5 exposure on some cognitive tests, but not consistently for all cognitive outcomes. These findings contribute to emerging evidence that elevated levels of indoor PM2.5 may affect not only physical health outcomes, but also that have broader effects on cognitive performance, productivity, and potentially mental health—although not all cognitive abilities may be equally affected by exposure.

For context, our laboratory exposure level (31.2 μg/m^3^) closely matched the global average PM2.5 exposure of 31.3 μg/m^3^ reported in the State of Global Air 2024, although the latter represents yearly averages across the world.^5^ This lab study builds on a previous field experimental study^22^^,^^23^ conducted by the authors, which found similar results with average PM2.5 levels of 18.0 μg/m^3^ and 3.7 μg/m^3^ in the control and intervention condition, respectively. Notably, average PM2.5 levels in our high-exposure conditions—31.2 μg/m^3^ in this laboratory work and 18.0 μg/m^3^ in the field work^22^^,^^23^—exceeded the recently proposed indoor air quality guideline of 15 μg/m^3^ for public buildings by a group of air quality experts.^43^ This recommendation adapts the WHO’s 24-h air quality threshold of 15 μg/m^3^ but applies it as a 1-h average for indoor environments. Therefore, there is an urgent need for improved indoor air quality management in public buildings, as over 95% people worldwide are routinely exposed to PM2.5 levels that exceed recommended WHO health-based guidelines,^5^ potentially impacting their cost of cognitive performance and overall well-being as illustrated in this study.

This lab study and our previously published field studies,^22^^,^^23^ while independent, are highly complementary and provide robust evidence on the impacts of short-term indoor PM2.5 exposure on cognitive skills, particularly in memory and attention. These findings align with several previous studies, most of which were epidemiological observational studies that investigated similar associations but focused on a limited range of cognitive domains. For example, a longitudinal observational study for office workers in multiple urban offices, found that indoor PM2.5, particularly above 12 μg/m3 (US annual ambient air quality standard), was associated with slower reaction times and reduced accuracy in the Stroop task and math test.^18^ Another study involving 30 university students^17^ found that 1 h of exposure to candle burning (with PM2.5 levels averaging 41.4 μg/m^3^, SD 46.1) linked with declines in MMSE and attention test scores, compared to a condition without candles burning (mean PM2.5: 1.6 μg/m^3^, SD 1.3). Additionally, research has linked both daily and hourly exposure to wildfire smoke with reduced attention performance in brain training games.^19^ Furthermore, longer-term exposure studies have also frequently reported cognitive effects in previous.^12^^,^^13^^,^^14^^,^^15^ For instance, an observational study in a male veterans-cohort found that 28 days of PM2.5 exposure was associated with decline in MMSE score and cognition.^10^ It should be acknowledged that cognitive effects may be influenced by exposure thresholds and population characteristics as suggested by other studies,^10^^,^^18^ whereby Gao et al., (2021)^10^ focused on older retired male population (>70 years) and on 28 days exposure.

The magnitude of cognitive effects found in this study could also be considered in the context of practical applications, e.g., productivity in the workplace. We found that the exposure to high levels of PM2.5 showed relatively consistent impacts on memory and attention domains, mixed effects on the perception domain, and no effects on the coordination and the reasoning domain. Considering cognitive performance as proxy of work performance,^44^ productivity might be differentially impacted by PM2.5 depending on the degree with which these cognitive skills are needed for a specific job-related tasks and overall occupation. Therefore, our findings may help to explain why previous studies on PM air pollution exposure and productivity in different sectors showed slightly different findings. For instance, exposure to PM significantly decreased productivity in the stock trading sector^45^ and among call center workers,^46^ but no significant associations were found in the manufacturing outputs of textile industry workers.^47^ The adverse effects of PM2.5 exposure on memory and attention may impact productivity in sectors like healthcare, engineering, education, legal fields, arts, and management, as ranked by the *Occupational Information Network (*O∗NET) database^48^ based on the importance of these cognitive functions. It should be acknowledged that converting the results of cognitive tests into predictions of actual productivity is an open question and is complex and context-dependent. For example, a reaction time difference of 50–100 ms is more critical when driving or at the controls of an aircraft, and in decision-making in high-skilled, quality-focused occupations.^49^ PM2.5 has been associated with increased traffic accidents,^50^ illustrating the potential practical implications of these cognitive effects. The statistically significant reaction-time differences which were found in this study ranged from about 37 to 581 ms depending on tasks, with small-to-moderate effect sizes, and the global cognitive score showed a moderate effect (Cohen’s d = 0.60). Although relatively small for an individual response, such differences may accumulate during frequently repeated workplace tasks and may be relevant to activities requiring rapid attention and decision making, such as in driving.^51^ Illustratively, a delay of 37–203 ms would correspond to approximately 0.5–2.8 m of additional travel at 50 km/h in driving. Nevertheless, the study did not directly assess work productivity, driving safety, or clinical impairment; therefore, these examples indicate potential functional relevance rather than established real-world or clinical effects.

Our lab study showed HRV indices were higher during cognitive testing in the low PM2.5 exposure condition, indicating higher cardiovagal relative to sympathetic control on the heart, which is related to better cognition and stress resilience. The largest effect size of PM2.5 level was found for RMSSD, which is considered to be more specific to cardiovagal control compared to SDNN and pNN50.^40^ We found the same pattern of the HRV indices during the cognitive testing in our previous field study^23^ strengthening the external validity of the lab study findings. This association between PM2.5 and HRV during cognitive testing persisted after adjusting for the pre-cognitive testing HRV score and sex. Existing studies have shown mixed results on the effect of indoor air filtration interventions on the autonomic nervous system as measured by HRV.^52^ Specifically, a three-day intervention in a care home observed a non-significant inverse association between PM2.5 and HRV in 40 older adults,^53^ and a non-significant positive association was observed in a two-week intervention in senior living residences.^54^ One randomized controlled trial (RCT) reported a significant decrease in HRV during the use of an ionization air purifier,^55^ which may reflect an adverse cardiovascular effect of the ozone produced by electrostatic and ionization air purifiers.^52^ Another study found no significant changes in HRV outcomes with the use of air purifiers in 38 college students in student accommodation but observed significant changes in blood pressure and blood oxygen saturation.^56^ However, none of those studies measured HRV while participants were engaged in a cognitively demanding condition.

Higher HRV indices are consistently associated with better cognitive outcomes and mental health,^33^^,^^57^ and increasing evidence supports a bidirectional relationship with the integrity of prefrontal self-regulatory regions.^58^^,^^59^ To date, the pathways and mechanisms underlying PM-associated cognitive dysfunction are poorly understood.^2^ Emerging evidence supports associations between air pollution—such as PM2.5 exposure—and cognitive dysfunction through various pathways and mechanisms mostly focus on long-term exposure including neurotoxicity, neuroinflammation, oxidative stress, and damage in blood-brain barrier and neurovascular units.^60^^,^^61^ Recent experimental evidence^21^ has significantly contributed to our understanding of the cognitive impacts of acute PM exposure, suggesting that attention skills may be impaired after 4 h exposure through lung-brain mechanisms—either via direct translocation or indirect systemic responses—though not necessarily mediated by inhalation patterns alone. Notably, our findings extend current knowledge by showing that reductions in cardiac autonomic regulation, indexed by reduced HRV, mediate the association between short-term PM2.5 exposure and cognitive performance decline. These findings may support a “bottom-up” neurovisceral integration pathway (reflecting heart-brain communication mechanisms),^29^^,^^30^ wherein PM2.5 exposure disrupts autonomic homeostasis and may, in turn, contribute to cognitive decline. Although the mediation findings suggest that HRV may represent a potential physiological pathway linking PM2.5 exposure with cognitive performance, we acknowledge that HRV and cognitive performance were assessed concurrently during cognitive testing; therefore, causal directionality cannot be fully assumed.

The absence of significant EDA effects alongside the HRV findings may reflect differences in the physiological processes captured by these measures. EDA, a measure of sweat-induced changes in skin conductance, is proposed to index sympathetic control^28^ and should not be considered a comprehensive measure of systemic sympathetic activity. By contrast, HRV indices examined here predominantly reflect cardiac vagal modulation.^29^^,^^30^ Thus, the findings do not necessarily indicate unaffected sympathetic activity alongside altered parasympathetic activity, but rather reflect differences in the sensitivity and physiological specificity of these measures. EDA may also be less sensitive to the type of sustained, low-level environmental stressor examined here because it is particularly responsive to emotional, thermal and salient arousal events.^28^ The controlled temperature and relative humidity, participant blinding, and absence of an acute emotional stimulus may therefore have reduced differences in EDA between conditions. Accordingly, the null EDA findings might be interpreted as an absence of detectable effects on electrodermal indices of sympathetic sudomotor activity, rather than evidence that sympathetic nervous activity as a whole was unaffected.

This study mainly focused on working-age adults with most participants aged 18 to 30 years; future studies should investigate wider age ranges, including mid-life and older adults with and without cognitive impairment. Although CO_2_ concentrations were statistically different between the two conditions, the absolute difference was small, approximately 63 ppm (873.2 versus 809.8 ppm). A difference of this magnitude in CO_2_ is considered unlikely to have a practical impact on health, comfort or cognitive performance^35^^,^^36^ Furthermore, sensitivity analyses additionally adjusting the cognitive models for measured CO_2_ showed the cognitive findings remained statistically significant (Table S4). The fine PM in this study was mostly PM1, defined as particles with an aerodynamic diameter of ≤1 μm, which is the main contributor to PM2.5. This is consistent with previous real-world monitoring studies showing that PM1 accounts for a significant proportion (70%–80%) of personal exposure to PM2.5 across various settings in adults.^62^^,^^63^ We use PM2.5 mass concentration as our metric of PM2.5 pollution, following standard public health protocols.^34^ Additionally, the PM2.5 generated by candles combustion in this study may have unique chemical components of air pollution. However, the PM2.5 in this study was generated by non-scented candle combustion, and an identical candle-burning protocol was used under both exposure conditions, with the primary experimental difference being the use of a HEPA filter or sham filter. Candle-related gaseous co-pollutants would therefore be expected to have been consistently present in both conditions, while the particulate fraction was substantially reduced in the low fine PM condition. This is supported by the absence of a significant difference in measured TVOC concentrations between conditions. Nevertheless, detailed chemical speciation was not undertaken, and possible effects of unmeasured particle-bound compounds cannot be excluded. Accordingly, the findings are most directly generalizable to exposure to indoor combustion-derived fine PM and to particle-reduction interventions, rather than implying identical effects across all PM2.5 sources. However, consistency with previous studies^10^^,^^20^^,^^21^ involving mixed-source indoor PM, ambient pollution, and wildfire smoke supports the broader relevance of the observed cognitive and autonomic responses. In addition, the PM2.5 source by combustion could represent the typical source of PM in real-world, as previous research^64^ showing that global PM2.5 exposure from combustion sources imposed more than US$1 trillion in health costs in 2019, representing 56% of the total health costs from all PM2.5 sources. Notably, combustion sources contributed a higher share of health costs than their proportional contribution to population-weighted PM2.5 concentrations in 134 countries, covering over 87% of the global population (ibid). Research to date has considered different exposure conditions, varying in duration, PM2.5 concentration levels and source type on cognitive effects.

In dose-response sensitivity analyses, we further examined whether within-condition variation in PM2.5 concentration was associated with global cognitive score. These analyses were performed separately within the high and low PM2.5 exposure conditions and were adjusted for sex and CO_2_ (Figure S3). The associations were not statistically significant (*p* > 0.1). These findings suggest that the relatively small variations in PM2.5 concentration within each controlled exposure condition were not associated with global cognitive score. This may reflect the relatively well controlled range of PM2.5 concentrations within this experimental study in the chamber, compared with the much larger exposure variations in epidemiological studies. Future research may consider study designs, which aim to further disentangle these aspects such as dose-response relationship in adult populations.

In conclusion, our work demonstrated the cognitive harms of indoor short-term particulate air pollution exposure and appraised potential mechanisms. Consequently, improving indoor air quality by reducing PM pollution could reduce not only adverse physical health outcomes, such as morbidity and mortality but also have broader impacts such as aspects of cognitive performance. This finding highlights the importance of focusing on strategies to mandate indoor air quality to achieve greater health, wellbeing, and productivity benefits.

### Limitations of the study

Physiological data were unavailable for 11 participants because the participants reported that recent ear piercing made earlobe PPG sensor attachment infeasible. Future studies using earlobe PPG sensors may include recent ear piercing in the pre-screening checklist. There was no statistical evidence of a difference in sex distribution between participants without physiological data and those with physiological data (8/11, 72.7%, versus 50/79, 63.3%, Fisher’s exact *p* > 0.1). Age-band and educational distributions were also similar between the two groups (both Fisher’s exact *p* > 0.1; Table S3). The available sample of 79 participants retained 92.8% power and the exposure comparison was within-participant in a randomized crossover design, and all analysis models were adjusted for sex, reducing the potential influence of the small difference in sex composition. The study was designed to estimate the within-participant effects of the PM2.5 exposure condition and accordingly was not designed to assess sex as an effect modifier and the unequal numbers of male and female participants limited the precision of potential sex-by-exposure estimates. Larger studies with prespecified design are needed to determine whether responses to short-term fine PM exposure differ by sex in terms of health, cognitive performance, and productivity.

## Resource availability

### Lead contact

Requests for further information and resources should be directed to and will be fulfilled by the lead contact, Jiaxu Zhou (jiaxu.zhou.19@alumni.ucl.ac.uk).

### Materials availability

This study did not generate new unique reagents.

### Data and code availability

• This paper does not report original code.
• Data reported in this paper have been deposited at Mendeley Data (DOI: https://data.mendeley.com/datasets/hxph9n9w7v/1) and are publicly available as of the date of publication.
• Any additional information required to reanalyze the data reported in this paper is available from the lead contact upon request.

## Acknowledgments

The authors gratefully acknowledge support from the BSEER Student Development Fund (SDF). K.L. is funded by the 10.13039/501100000265UK Medical Research Council (MR/S021418/1).

Thanks to Prof Dejan Mumovic for his guidance and support on the application of the Advanced Air Quality and Thermal Studies Lab (UCL Here East); to Prof Hector Altamirano and Dr Samuel Stamp for their technical support for the chamber and monitors; to Prof Peter Raynham for his kind guidance and support on risk assessment for this work; and to participants for their continuous participation.

## Author contributions

J.Z.: writing – original draft, visualization, validation, software, resources, methodology, investigation, formal analysis, data curation, conceptualization. G.H.: writing – review and editing, validation, supervision, methodology, investigation, formal analysis, conceptualization. K.Y.L.: writing – review and editing, validation, resources. M.U.: writing – review and editing, validation, supervision, resources, project administration, methodology, investigation, funding acquisition, conceptualization.

## Declaration of interests

The authors declare no competing interests.

## STAR★Methods

### Key resources table

**Table:** 

REAGENT or RESOURCE	SOURCE	IDENTIFIER
**Deposited data**		

Mendeley Data and Code	This paper	https://data.mendeley.com/datasets/hxph9n9w7v/1

**Software and algorithms**		

R software version 4.3.1	R Core Team	https://www.r-project.org/

### Experimental model and study participant details

#### Study design

The study employed a randomized single-blind crossover design with 90 participants. Using a counterbalanced approach, each participant individually experienced both High and Low PM2.5 exposure conditions in the chamber, with each exposure lasting 1.5 h. A repeated-measures design was used, with endpoints testing assessed twice—once in each condition— the two experimental sessions were separated by a one-week washout period.

#### Ethics, data protection and risk assessment

The study was approved by the institutional review board of University College London (UCL), Bartlett School of Environment Energy and Resources, with the registration number for Ethics (No. 20230324_IEDE_PGR_ETH). It also adhered to UCL data protection rules, as indicated by Data Protection (No. Z6364106/2023/03/124). The risk assessment was authorized under reference RA071116/1. All participants gave written informed consent ahead of the study.

#### Participants and sample size

This study involved 90 participants based on a sample size calculation using G∗Power software. We set α = 0.05 and power (1−β) = 0.95, with a small effect size f^2^ = 0.15, which corresponds to d = 0.30^65^ as observed in the previous literature.^66^ The calculation was based on linear multiple regression analysis with two fixed predictors: one for PM2.5 experimental condition and the other to accommodate potential analyses of demographic variables (e.g., sex differences) on the test endpoints. The analysis indicated a required sample size of 89 participants to detect the expected effect. A total of 90 participants were completed in the study, meeting the calculated sample size requirement (See Figure S1).

Participants were recruited from university students and staff through email and advertisements. Inclusion criteria were: being aged between 18 and 65 years, not smoking, being healthy without chronic long-term conditions, not using prescription medications and antiarrhythmic drugs (including beta-blockers, calcium channel blockers, digitalis etc.), and having no mental and learning disorders. Participants were paid for their participation with a £20 voucher once they finished the whole experiment.

Physiological data (HRV and EDA) was collected from 79 out of the 90 recruited participants. This was because HRV recording required attachment of a photoplethysmography sensor to the earlobe, and these 11 participants reported recent ear piercing at the time of testing, making sensor attachment uncomfortable or infeasible. However, maintaining the consistency of the experimental testing interval across all participants was the priority, thus it was decided not to collect physiological data from these 11 individuals. Recent ear piercing was not included in the original pre-screening checklist, as it was not anticipated to occur frequently and did not affect eligibility for the cognitive or exposure components of the study. Information on why participants had recently had their ears pierced was not collected, as this was not part of the ethics-approved study protocol. The absence of the physiological data was therefore due to measurement feasibility rather than post-hoc data exclusion or signal-quality filtering. In addition, comparisons between participants with and without physiological data showed no statistical significant evidence of imbalance in demographics (*See* Table S3). A sensitivity power analysis based on the final physiological sample of 79 participants and the prespecified assumptions used in the original study planning indicated a sufficient power of 0.928 to detect a statistical difference (*See* Figure S1). The results are reported with 95% confidence intervals and statistical significance was evaluated at α = 0.05 in this study.

### Method details

#### Chamber features and settings

The lab work was conducted in a stainless-steel environmentally controlled chamber approx. 60 m^3^, with 4.5 m wide × 4.5 m deep × 3.0 m high (Figure S2) located at the university premises in East London, UK. The climate chamber was set up to create stable indoor environmental parameters, reflecting conditions that are recommended for real office buildings: air temperature (23°C), relative humidity (50%), and ventilation rates (108 m3/h,1.8 ACH, 30 L/s-p). In addition, the chamber environment has an air circulation system through four fans to ensure the indoor air is well-mixed. The air supply was brought into the chamber through a building ventilation system equipped with a HEPA filter.

#### Procedure

During each session, physiological response monitoring was collected before and during cognitive testing, measuring heart rate variability (HRV) and electrodermal activity (EDA). In addition, the weekday and timing of each individual’s experiment session was kept consistent across both conditions (e.g., 1^st^ session at Monday 1.00 p.m., 2^nd^ session at next Monday 1.00 p.m.). Participants were informed to wear the same clothes for the two sessions. In addition, the high exposure and low exposure test sessions were counterbalanced within each day, such that neither condition was systematically conducted earlier or later in the day, to ensure that any variation in background environmental levels did not have an undue influence. Detailed information on the stopping procedures, fire precautions and adverse-event monitoring is provided in Methods S1*: participant safety procedures.*

#### Exposure condition

There were two conditions: High and Low PM2.5 exposure condition. Both conditions involved burning three new non-scented paraffin candles (petroleum wax) in a fireproof holder as a source of PM2.5 air pollution. The candles burnt for 30 min before participants entered the chamber to achieve a stable PM2.5 level. When participants were in the chamber the candles were kept burning. The number of candles used was chosen based on a target level of 25–30 μg/m3 of PM2.5 and maintaining a stable concentration for 2 h. This level is representative of most public spaces^17^^,^^67^^,^^68^ and aligns with the average global PM2.5 exposure of 31.3 μg/m^3^, as reported in the State of Global Air 2024 report.^5^

We used Philips AC5659/10 air purifier in both conditions, located in the center of the chamber, with the HEPA filter during the Low condition and a sham one during the High condition. The HEPA air purifier has a clean air delivery rate (CADR) of 500 m^3^/h with a 0.3 μm particle removal efficiency of 99.97%, for room sizes up to 60 m^2^. The other environmental parameters such as ventilation rate (108 m^3^/h, 30 L/s-p), temperature (23 °C) and relative humidity (50%) were kept constant in both conditions. The ventilation rate was set at 108 m^3^/h (30 L/s-p), based on findings from previous research conducted using the same chamber. This rate was determined to maintain CO_2_ levels generated by a single occupant at relatively low concentrations, approximately 600–700 ppm.^69^ The temperature of 23 °C was chosen to correspond to the recommended temperature for public buildings 20.5°C–25.5 °C in ASHRAE Standard,^70^ and the relative humidity of 50% corresponded to the recommended relative humidity of 40%–70% by Health and Safety Executive UK. They also match recommendations for a comfortable environment from previous studies^36^^,^^69^ and our fieldwork in Beijing.^22^^,^^23^^,^^71^

#### Measures

##### Exposure assessment

The indoor environment was monitored using dedicated instruments. The DustTrak DRX aerosol monitor (TSI-8533) was employed specifically to track indoor PM2.5 levels continuously. Additionally, environmental parameters were continuously measured including indoor CO2 levels, temperature, and relative humidity within the chamber. A portable monitor -TSI 7575 instrument-was placed at a height of 1.2 m within the center position of the chamber to monitor indoor CO_2_ levels, temperature, and relative humidity. A PhoCheck Tiger PID monitor continuously measured the TVOC levels. All monitors were set to a 1-min monitoring interval and were calibrated before the experiment by manufacturers. The technical specifications of these monitors, as provided by their respective manufacturers, are illustrated in Table S1 for reference.

##### Cognitive performance assessment

Cognitive performance was tested with the commercially available computer-based neurological battery test General Cognitive Assessment Battery, CogniFit (CogniFit Inc., San Francisco, USA). The assessment covered a wide range of cognitive domains including memory, attention, perception, coordination, and reasoning. In each domain, various sub-skills were assessed, with 16 skills in total included. Task metrics considered for the analysis were: reaction time of an accurate response in milliseconds, reaction time of any response for tests where no accuracy was assessed in milliseconds, and, where available depending on the task, accuracy in percentage and correct answers.

The General Cognitive Assessment Battery evaluation has been validated in healthy people against major standard neuropsychological tests, including the full Cambridge Neuropsychological Test Automated Battery, Raven’s Standard Progressive Matrices, the Wisconsin Card Sorting Test, the Continuous Performance Test, the STROOP test, and other tests.^72^ Tests of its reliability have been demonstrated in previous studies, yielding adequate measures of internal consistency (Chronbach’s alpha = 0.70) and test-retest reliability (intra-class correlation coefficient = 0.80)^38^^,^^72^ It has been used as a cognitive function measure in previous research.^73^^,^^74^^,^^75^^,^^76^^,^^77^ The cognitive tasks details and specifications have been reported previously in our fieldwork.^22^ A brief description is available in the Table S2.

In addition, the ‘Cognitive Assessment Battery’ uses a proprietary algorithm that analyses a large number of variables to provide global cognitive scores as percentile ranks (multiplied by 8), which conform to a normal distribution. According to the Cognifit manual, the global cognitive scores range from 0 to 800 with higher scores indicating better cognitive performance.

##### HRV and EDA assessment

HRV and EDA responses were monitored using wireless wearable sensors (ErgoLab, King-far Inc., Beijing, China). HRV was calculated according to the guidelines of the Task Force of the European Society of Cardiology^78^ including Time Domain Metrics (standard deviation of normal-to-normal intervals [SDNN]; root-mean-square of the successive differences [RMSSD]; percentage of successive R-R intervals that differ by more than 50 ms [pNN50]), and Frequency Domain Metric (high-frequency spectral power [HF]). EDA was measured by the changes in skin conductivity due to sweat glands’ activity, which consisted of two components: the phasic component (Skin Conductance Response [SCR]) and the tonic component (Skin Conductance Level [SCL]).

### Quantification and statistical analysis

Cognitive test data cleaning was done separately for both conditions and separately for each task. The raw data of the cognitive tasks was z-transformed prior to use in analyses. If the z value was outside the range from −3 to 3, it was considered an outlier and hence excluded. All HRV and EDA outcomes were log10-transformed. The study tested whether short term exposure to indoor PM2.5 affected cognitive performance, HRV and EDA outcomes. The analysis utilized a Generalized Linear Mixed Effect Regression Model with PM2.5 exposure condition and sex as categorical fixed effects. Participants code is coded as a random effect intercept to account for correlations of repeated measurements per subject. The model was formulated as:

$$Y_{\text{ic}}=\beta_{1}*\;Condition+\beta_{2}*\;Sex+v_{i}+\varepsilon_{\text{ic}}$$

where Y_ic_ represented cognitive task metrics of individual i measured at condition c; β1 and β2 were fixed-effect coefficients respectively for experiment conditions and sex; vi was a random-effect intercept at individual level for individual i; and ε_ic_ was a normally distributed residual term that represented the random variation of individual i at condition. In addition, for variables with significant effects, Cohen’s d was calculated as an effect size indicator.^65^ An assessment of carry-over effects using the Global Cognitive Score was implemented. For each participant, scores from the two sessions were summed and the totals were compared between the high–low and low–high exposure-sequence groups using an independent *t* test.

We conducted a causal mediation analysis of the PM2.5-cognitive function relationship mediated by the cardiac response of HRV, by using a bootstrapping approach (1000 samples). We estimated Average Causal Mediation Effects (ACME), Average Direct Effects (ADE), and Total Effect. A bootstrap 95% CI for the specific average causal mediation effect, did not include zero providing evidence for statistically significant mediation.

All statistical significance was evaluated at α = 0.05 in this study. Multiple hypothesis testing corrected by false discovery rate (FDR) correction,^37^ the FDR-adjusted *p*-value smaller than 0.05 was considered as statistical significance. Statistical analyses of cognitive and physiological outcomes were performed using R software^79^ with the ‘lme4’ package for the mixed effect model,^80^ and the ‘mediation’ package for mediation analysis.^81^

## Supplemental information

Document S1. Figures S1–S3, Tables S1–S5, and Method S1
