# Supplementary material for: Short-term indoor fine particulate matter exposure impedes cognitive performance and autonomic nervous system in adults
Source: iScience. 2026 Oct 15;29(11):117904. doi: 10.1016/j.isci.2026.117904 (PMC13694848; doi:10.1016/j.isci.2026.117904)
Supplement: Supplementary file 1 [file mmc1.pdf]

iScience, Volume *29*

Supplemental information

Short-term indoor fine particulate matter exposure impedes cognitive performance and autonomic nervous system in adults

Jiaxu Zhou (周家绪), Gesche Huebner, Kathy Y. Liu, and Marcella Ucci

Supplementary Material

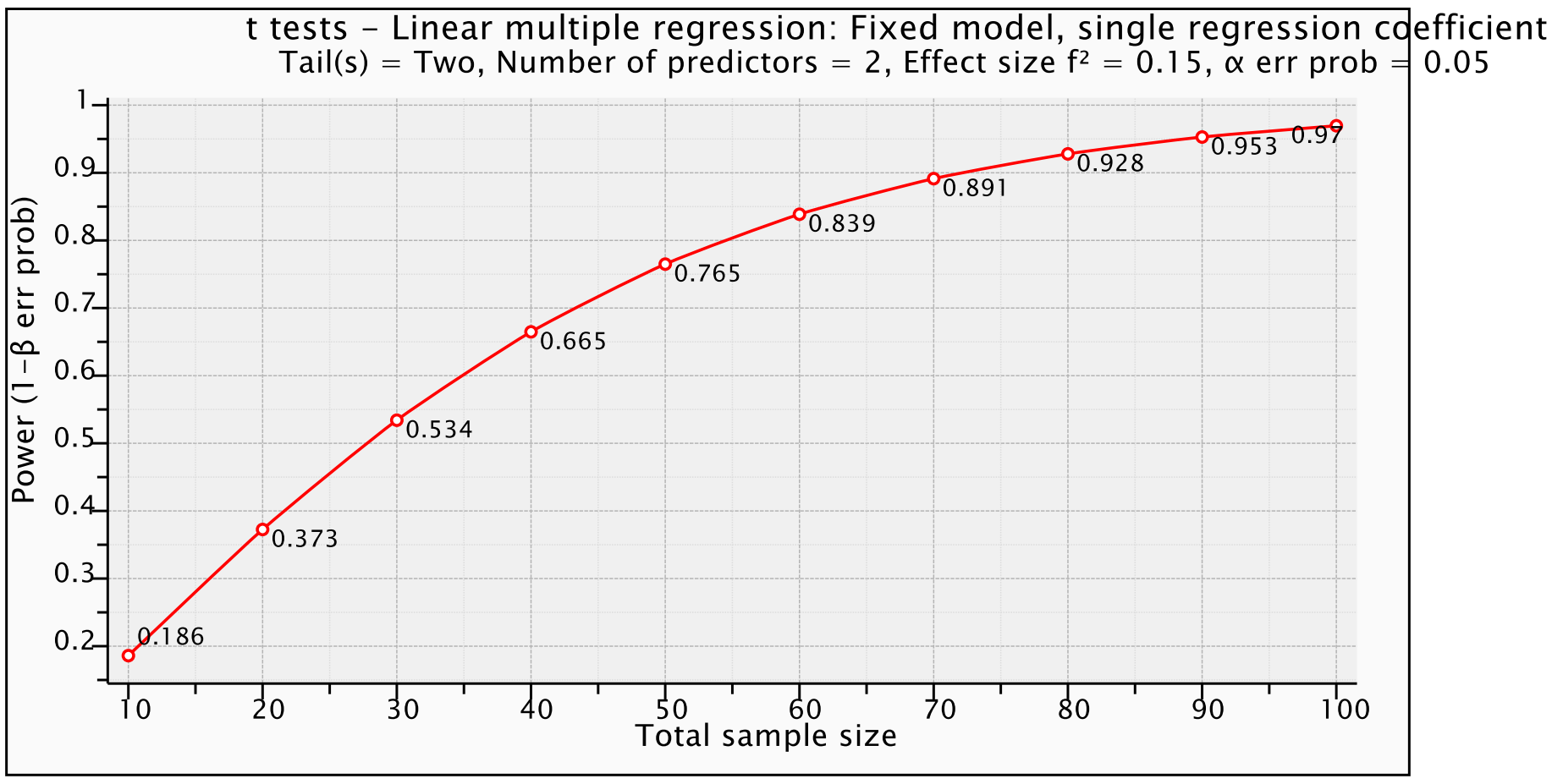

Fig S1. *Sample size calculation of the lab work and sensitivity check*

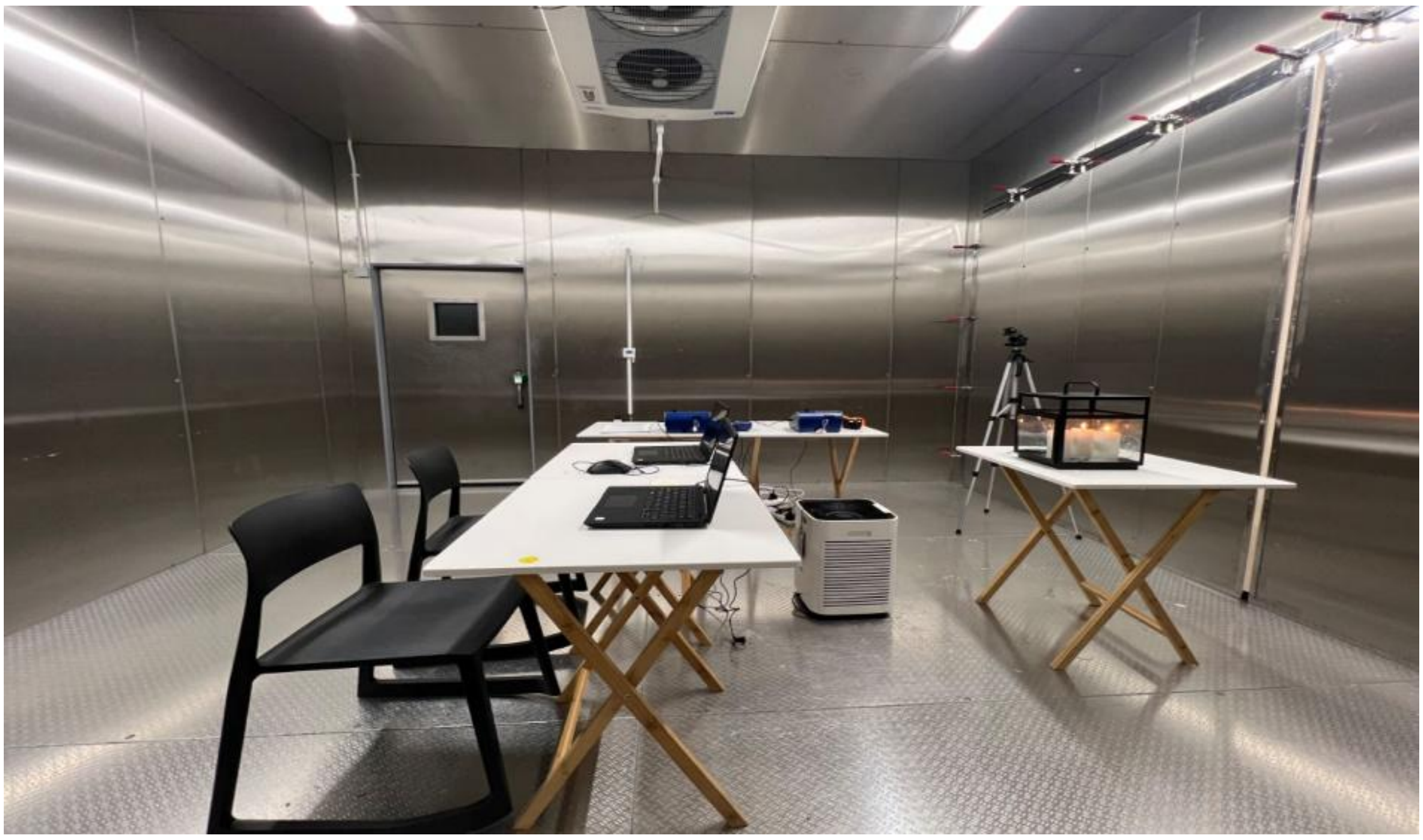

Fig S2 *Chamber setting*

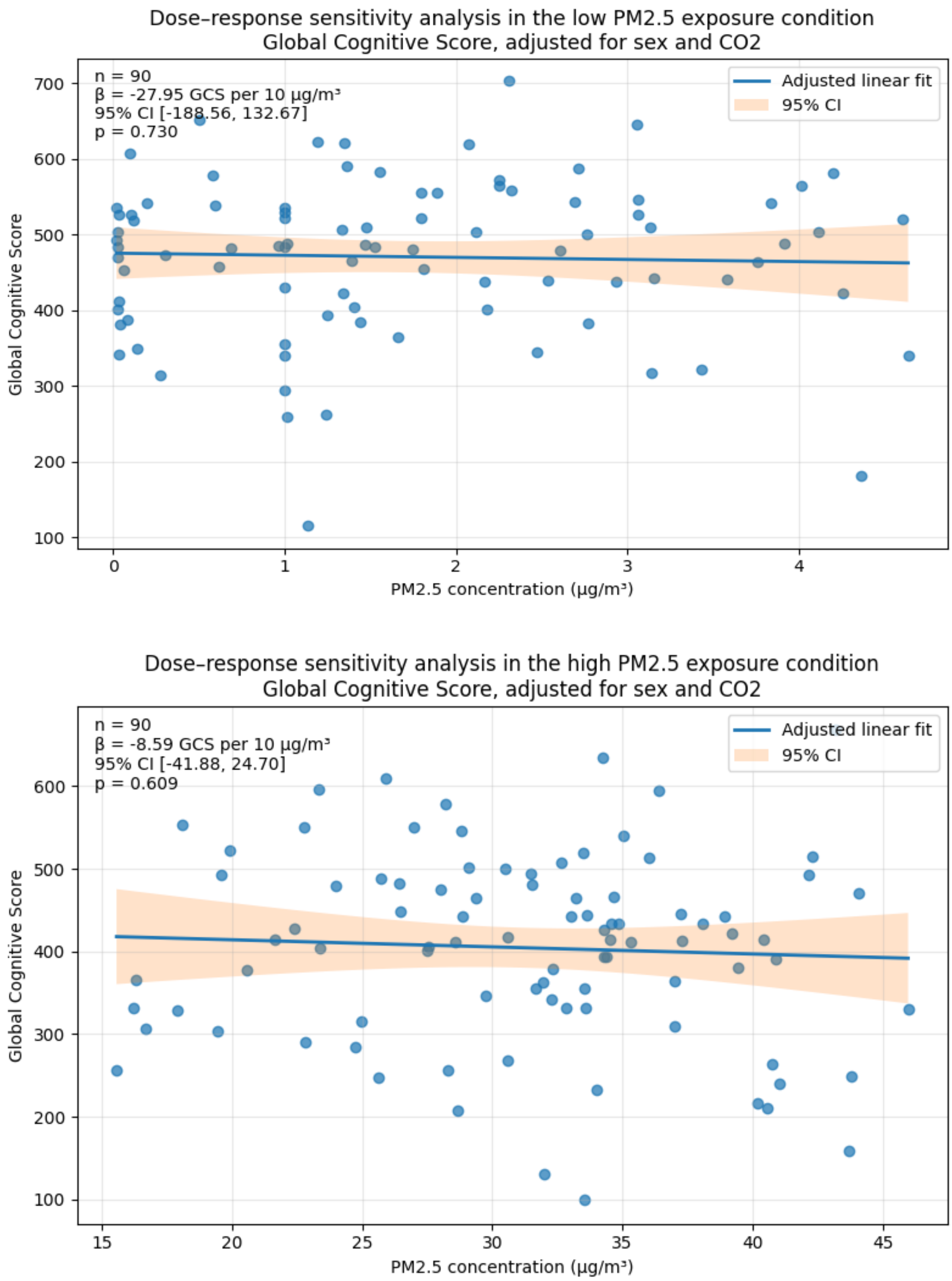

Fig S3. *Dose–response sensitivity analyses between PM2.5 concentration and Global Cognitive Score within the high and low PM2.5 exposure conditions. Linear fits are adjusted for sex and* CO_2*; shaded areas represent 95% confidence intervals.*

Table S1. *Technical specifications of all monitors used in this study*

Monitors	Measurement	Specifications
TSI 7575	CO_2	Measurement range: 0-5000 ppm<br>Accuracy: ±3% of reading or ±50 ppm, whichever is greater ppm<br>Resolution: 1 ppm
	Temperature	Measurement range: 0-60 ˚C<br>Accuracy: ±0.5˚C<br>Resolution: 0.1 ˚C
	Relative Humidity	Measurement range: 5-95%<br>Accuracy: ±3%<br>Resolution: 0.1 %
PhoCheck Tiger	Total Volatile Organic Compounds (TVOC)	Measurement range: 0-20000 mg/m^3<br>Accuracy: ±5% display reading
DustTrak DRX model TSI 8533	Particulate Matter (PM): PM1, PM2.5, PM10, PM total	Measurement range: 0.001-150 mg/m^3<br>Resolution: ±0.1% display reading, or 0.001mg/m^3 which ever is greater

Table S2. *Cognitive task description*

Domain	Skill	Task	Brief Description
Memory	Phonological Short-term Memory	Objects Seen or Heard Before	Memorize the presented objects and their presentation method. Then, indicate if the last presentation was an image, sound, or if it's the first time you've seen the object.
	Contextual Working Memory	Pictures and Words	The test-taker sees a series of objects. In a new series, they must recognize previously displayed objects, which can be presented as images or spoken words.
	Short-Term Memory	The Numbers	Memorize an increasing amount of numbers in each series to repeat them in the same order.
	Visual Short-Term Memory	Glowing Circles	Stimulus are displayed irregularly across the screen. They will light up in a random order, and the user need to memorize the sequence.
	Naming	The Letters	A series of images will be displayed that the user must identify and associate with their corresponding name (naming).
Attention	Divided Attention	The Ball and the Colours	The test-taker is required to accurately follow a ball moving and turning in all directions on the screen while, at the same time, performing a variant of the Stroop test.

	Inhibition	The Words and the Colours	The test-taker needs to perform a Stroop test
	Focused Attention	The Circles and Hexagons	The test-taker is required to rapidly press on circles which appear on the screen and to ignore hexagons should they also appear.
	Updating	Numbers and Shapes	First, click the larger figure, ignoring numbers and words. Then, click the figure with the higher number, ignoring size.
Perception	Auditory Perception	Musical Notes	The task involves interrupting an auditory stimulus to match the length of a previously presented one
	Estimation	Fast and Curious	The test-taker estimates the speed, distance, and their interaction on a moving object's motion.
	Recognition	Three Shapes	Memorize a trio of figures. Identify the identical trio from four options after the original trio is removed.
	Visual Scanning	The Circles and Hexagons	Rapidly press circles appearing on the top right of the screen and ignore any hexagons.
Coordination	Hand-eye Coordination	Follow the ball	The test-taker carefully and precisely track a ball moving along a path.
	Response Time	A big Circle	Click the mouse as many times as possible within the given time and allowed area.
Reasoning	Planning	The Mazes	Reach the goal of each maze in the shortest possible time and with the fewest possible steps.

Table S3. *Demographic characteristics of participants with and without physiological data*

Characteristic	Full cohort, N=90	Physiological data available, n=79	Physiological data unavailable, n=11	p-value
Sex				p>0.1
Male	3（35.6%）	29（36.7%）	3（27.3%）	
Female	5（64.4%）	50（63.3%）	8（72.7%）	
Age band				p>0.1
18–30 years	8（88.9%）	70（88.6%）	10（90.9%）	
31–40 years	10（11.1%）	9（11.4%）	1（9.1%）	
Education level				p>0.1
Bachelor’s degree	1（1.1%）	1（1.3%）	0（0%）	
Master’s degree or above	8（98.9%）	78（98.7%）	11（100%）	

p-values compare participants with and without physiological data using Fisher’s exact tests.

Table S4. *Sensitivity analysis of cognitive performance (n=90), with the low fine PM condition as the reference category. The β coefficients were adjusted for sex and* CO_2

Skill	Metric	β (95% CI)	p-value
Phonological Short-term Memory	Correct Reaction Time (ms)	207.69 (92.69, 323.86)	p<0.001
Phonological Short-term Memory	Accuracy (%)	-3.20 (-4.99, -1.36)	p<0.001
Contextual Working Memory	Correct Reaction Time (ms)	167.52 (33.47, 300.95)	p=0.016
Contextual Working Memory	Accuracy (%)	-0.56 (-4.53, 3.42)	p=0.781
Short-Term Memory	Correct Reaction Time (ms)	61.20 (18.68, 102.77)	p=0.005
Short-Term Memory	Correct Answers	-0.42 (-0.73, -0.12)	p=0.008
Visual Short-Term Memory	Correct Reaction Time (ms)	-10.94 (-47.32, 25.85)	p=0.559
Visual Short-Term Memory	Correct Answers	-0.32 (-0.62, -0.01)	p=0.043
Naming	Correct Reaction Time (ms)	313.65 (56.34, 569.25)	p=0.018
Divided Attention	Correct Reaction Time (ms)	34.76 (-21.39, 89.85)	p=0.222
Inhibition	Correct Reaction Time (ms)	125.86 (22.26, 228.65)	p=0.019
Inhibition	Accuracy (%)	-3.77 (-9.77, 2.30)	p=0.222
Focused Attention	Correct Reaction Time (ms)	12.30 (-44.25, 68.59)	p=0.670
Updating	Correct Reaction Time (ms)	48.66 (11.83, 85.08)	p=0.011
Auditory Perception	Accuracy (%)	-2.39 (-6.40, 1.67)	p=0.247
Estimation	Correct Reaction Time (ms)	523.88 (44.66, 1005.20)	p=0.035
Estimation	Accuracy (%)	-0.70 (-4.44, 3.03)	p=0.715
Recognition	Correct Reaction Time (ms)	-6.88 (-103.96, 91.21)	p=0.890
Visual Scanning	Reaction Speed (ms/pixels)	-0.07 (-0.30, 0.15)	p=0.534
Hand-eye Coordination	Accuracy (%)	-0.61 (-2.02, 0.81)	p=0.400
Response Time	Reaction Time (ms)	2.95 (-3.17, 9.05)	p=0.347
Planning	Accurate Answers	0.03 (-0.26, 0.33)	p=0.821

Table S5. *Statistical analysis of the cognitive performance in the experiment (n=90), with Low PM2.5 condition as the reference category. The β coefficients were adjusted for sex, and p-values were adjusted for the false discovery rate (FDR) using the Benjamini-Hochberg correction*

Skill	Metric	β (95% CI)	p-value	Adjusted p-value	Cohen's d
Phonological Short-term Memory	Correct Reaction Time (Milliseconds)	199.95 (105.80, 293.81)	<0.001	<0.001	0.455
	Accuracy (%)	-4.01 (5.50 -2.53)	<0.001	<0.001	0.650
Contextual Working Memory	Correct Reaction Time (Milliseconds)	154.83 (46.34, 263.48)	0.006	0.022	0.287
	Accuracy (%)	-2.50 (-5.92,0.91)	0.152	0.223	0.192
Short-Term Memory	Correct Reaction Time (Milliseconds)	43.60 (10.09,76.52)	0.011	0.026	0.128
	Correct Answers	-0.30 (-0.54, -0.05)	0.019	0.034	0.211
Visual Short-Term Memory	Correct Reaction Time (Milliseconds)	-13.29 (-42.63 15.91)	0.374	0.432	0.101
	Correct Answers	-0.44 (-0.69, -0.20)	0.001	0.003	0.379
Naming	Correct Reaction Time (Milliseconds)	368.94 (152.60, 586.31)	0.001	0.005	0.436
Divided Attention	Correct Reaction Time (Milliseconds)	61.16 (14.39,107.93)	0.012	0.026	0.324
Inhibition	Correct Reaction Time (Milliseconds)	203.03 (115.28, 291.40)	<0.001	<0.001	0.597
	Accuracy (%)	-7.01 (-12.18, -1.86)	0.009	0.024	0.364
Focused Attention	Correct Reaction Time (Milliseconds)	0.767 (-47.65, 49.18)	0.975	0.975	0.004
Updating	Correct Reaction Time (Milliseconds)	37.03 (7.03, 66.98)	0.017	0.034	0.233
Auditory Perception	Accuracy (%)	-2.25 (-5.53,1.04)	0.180	0.248	0.126
Estimation	Correct Reaction Time (Milliseconds)	581.18 (162.05, 997.59)	0.007	0.023	0.400
	Accuracy (%)	-0.69 (-3.96, 2.58)	0.677	0.709	0.061
Recognition	Correct Reaction Time (Milliseconds)	27.97 (-51.20, 107.10)	0.488	0.537	0.065
Visual Scanning	Reaction Speed (Milliseconds /pixels)	-0.10 (-0.29,0.10)	0.320	0.391	0.142
Hand-eye Coordination	Accuracy (%)	-1.09 (-2.27, 0.08)	0.071	0.120	0.221
Response Time	Reaction Time (Milliseconds)	4.36 (-0.64, 9.35)	0.089	0.140	0.166
Planning	Correct Answers (3 answers in total)	-0.17 (-0.42, 0.08)	0.195	0.252	0.156

Methods S1: participant safety procedures

Fire-safety precautions

Three new, non-scented candles were used in both experimental conditions. The candles were secured in a fireproof holder within a protected enclosure positioned in the corner of the chamber and away from the participant. The participant was seated closer to the chamber door than to the candles, allowing rapid exit if required.

Participants were not permitted to bring ignition sources or flammable materials, including lighters, alcohol or petrol, into the chamber. Portable fire extinguishers and water were available near the chamber. The researcher was able to stop the experiment, extinguish the candles and initiate evacuation through the designated fire-escape route if necessary. The chamber was also covered by the building's monitoring and alarm systems.

Experiment stopping procedures

A researcher continuously observed the participant and the chamber using a webcam connected to a separate computer. Two-way communication was maintained throughout the session. Participants could immediately inform the researcher if they experienced discomfort or wished to stop.

The researcher was authorized to terminate the session if a participant reported symptoms or discomfort, or if any other safety concern arose. The participant could then leave the chamber immediately and, if necessary, receive appropriate assistance.

Adverse events

No adverse events, exposure-related symptoms, early terminations, fires or other safety incidents were recorded during either experimental condition.
